# Association Between NAT2 Polymorphisms and Lung Cancer Susceptibility

**DOI:** 10.1097/MD.0000000000001947

**Published:** 2015-12-11

**Authors:** Chang Liu, Wei Cui, Lin Cong, Li Wang, Xinjian Ruan, Jia Jia, Yanfang Liu, Xiaoyan Jia, Xia Zhang

**Affiliations:** From the Department of Medical Oncology, The Military General Hospital of Beijing PLA, Beijing, China (CL, LW, XR, JJ, YL, XJ, XZ); Department of General Surgery, The Military General Hospital of Beijing PLA, Beijing, China (WC); and Department of Dermatology, The Military General Hospital of Beijing PLA, Beijing, China (LC).

## Abstract

To further investigate the association between *NAT2* polymorphisms and lung cancer susceptibility.

In terms of phenotypes, we investigated the acetylator status of *NAT2* polymorphisms associated with lung cancer risk. Additionally, in view of genotypes, we mainly analyzed 5 single nucleotide polymorphisms (SNPs) in *NAT2* gene, namely C282T, A803G, C481T, G590A, and G857A. Twenty-six eligible studies were included in our meta-analysis by searching PubMed, Embase, and CNKI databases. We used odds ratios (ORs) with corresponding 95% confidence intervals (CIs) to evaluate the susceptibility to lung cancer associated with *NAT2* polymorphisms.

Overall, based on phenotypes, the pooled ORs showed no significant association between *NAT2* polymorphisms and lung cancer susceptibility. In the subgroup analyses by ethnicity and source of control, there was still no significant association. In terms of genotypes, overall, no obvious relationship was observed between *NAT2* polymorphisms and lung cancer risk. But increased risk of lung cancer was found in association with *NAT2* C282T polymorphism (TT vs. CC + TC: OR = 1.58, 95% CI = 1.11–2.25).

Our meta-analysis demonstrates that TT genotype in *NAT2* C282T polymorphism may be a risk factor for lung cancer susceptibility. Additionally, the acetylator status of 5 SNPs in *NAT2* gene may not be associated with lung cancer risk.

## INTRODUCTION

Lung cancer is the most common cancer and the leading cause of cancer death in the world.^1,2^ It consists of 3 major histological subtypes, adenocarcinoma, squamous cell carcinoma, and small cell carcinoma. The exposure to tobacco smoke is known as a crucial cause of lung cancer.^3^ Additionally, genetic factors are considered to play an important role in lung cancer risk.^4,5^

*N*-acetyltransferase 2 (*NAT2*) gene, located on the short arm of chromosome 8 (8q22), encodes a phase II xenobiotic-metabolizing enzyme.^6,7^*NAT2* gene is essentially involved in the metabolism of aromatic, heterocyclic amines, and hydrazines.^8,9^ Five known polymorphisms in *NAT2* gene, namely C282T, A803G, C481T, G590A, and G857A, are associated with decreased enzyme activity and variable stability, leading to imbalance of the process of xenobiotics detoxification and consequently affecting lung cancer susceptibility.^10^

The alteration of NAT2 acetylator status caused by polymorphisms in *NAT2* gene may lead to decreased enzyme activity and absence of efficiency in detoxification, and further contribute to elevated cancer risk.^11^ There are 2 major NAT2 phenotypes, including rapid acetylator phenotype and slow acetylator phenotype. Wild-type homozygotes and heterozygotes in *NAT2* gene are categorized into rapid acetylator phenotype whereas mutant homozygotes are categorized into slow acetylator phenotype.^12^ The rapid acetylator phenotype was reported to increase the risk of bladder, colon, and prostate cancers.^13–15^ Whereas slow acetylator phenotype was in association with increased risk of bladder cancer and decreased risk of colon cancer.^16,17^

In 1995, Martinez et al first explored *NAT2* polymorphisms in malignancies among Caucasians. However, no significant association was observed.^18^ Since then, several phenotyping studies have investigated the association between acetylator status of *NAT2* polymorphisms and lung cancer risk.^12,19–38^ However, the results were inconclusive. With respect to genotypes, quite a few studies focused on the relationship between genetic polymorphisms in *NAT2* gene and lung cancer susceptibility.^39–42^ Therefore, we conducted this meta-analysis to gain more precise evidence for the association from genotype and phenotype aspects.

## METHODS

### Search Strategy

We searched PubMed, Embase, and CNKI databases using the terms “NAT2,” “polymorphism,” and “lung cancer.” The reference lists of the selected papers were also screened for other potential articles. The following inclusion criteria were used to select the eligible studies for this meta-analysis: case–control studies; enough data for estimating odds ratio (OR) with 95% confidence interval (CI). Additionally, when the same data were included in several publications, only the largest or most recent study was selected in our meta-analysis. All patients provided written or oral consent for participation in the registry, in accordance with local ethics committee requirements.

### Data Extraction

All the following data were independently extracted from each study by 2 investigators: single nucleotide polymorphisms (SNPs), first author, publication date, country of origin, ethnicity, source of controls, genotyping method, total cases and controls, and *P*-value for Hardy–Weinberg equilibrium (HWE), as shown in Tables 1 and 2. Inconsistent results were settled generally through discussion.

**TABLE 1:**
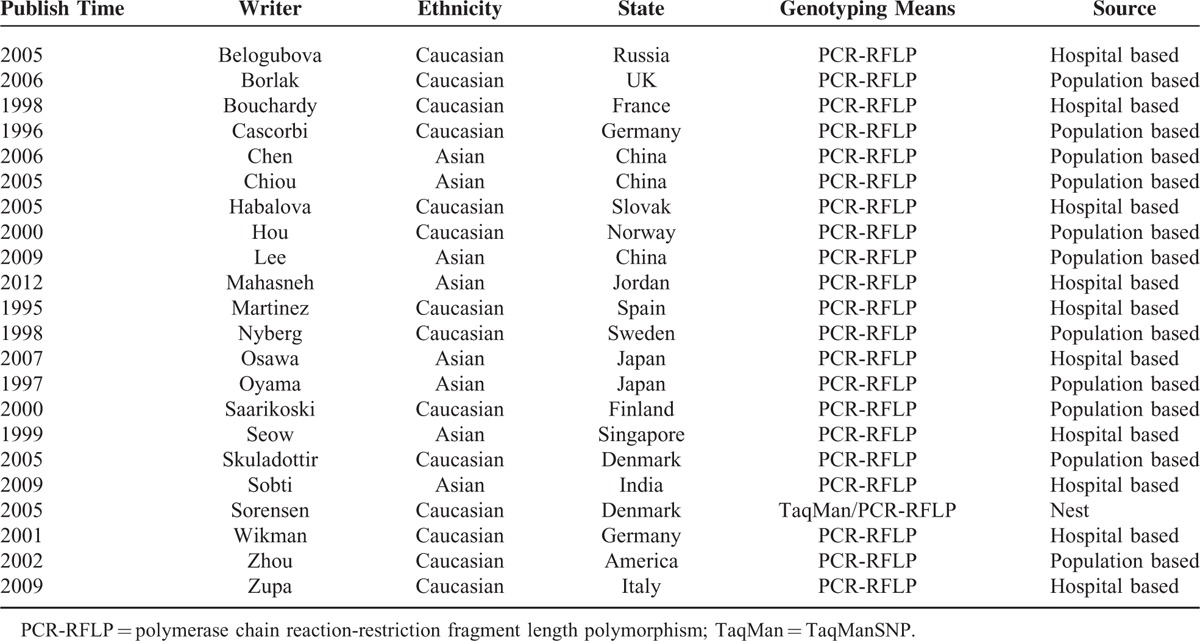
Principle Characteristics of the Studies Included in the Meta-Analysis Based on Phenotypes of *NAT2* Polymorphisms

**TABLE 2:**
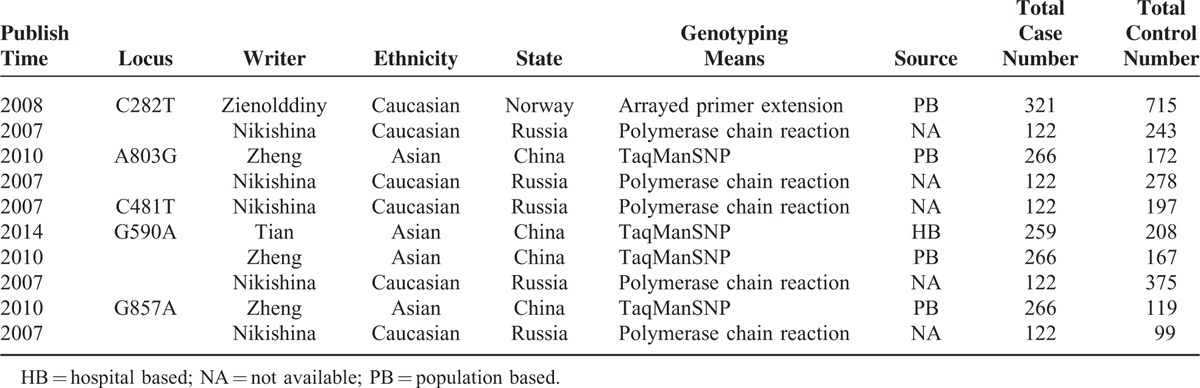
Principle Characteristics of the Studies Included in the Meta-Analysis Based on Genotypes of *NAT2* Polymorphisms

### Statistical Analysis

Crude ORs with 95% CIs were calculated to assess the strength of association between *NAT2* polymorphisms and lung cancer susceptibility. In terms of phenotypes, subgroup analyses were based on ethnicity and source of control. The Chi-square based Q-test was performed to evaluate heterogeneity. *P* < 0.05 indicates significant heterogeneity among studies, thus the pooled OR was calculated using random-effects model; otherwise, the fixed-effects model was used. Sensitivity analysis was performed to assess the stability of results. The potential publication bias was estimated by Egger test and Begg funnel plot. HWE was checked by χ^2^ test. Statistical analyses were conducted using the STATA software (version 12.0, Stata Corporation, College Station, TX).

## RESULTS

### Study Characteristics

**FIGURE 1:**
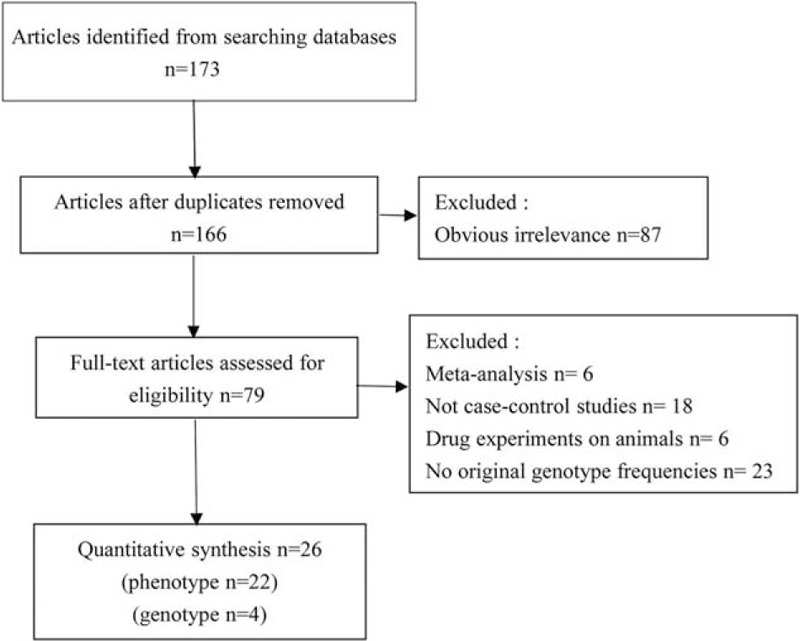
Flow diagram of included studies for the meta-analysis.

As displayed in Figure 1, a total of 173 studies were selected through databases in which 7 articles were excluded for duplicates and 87 articles were excluded for obvious irrelevance and finally 79 full-text articles were assessed for eligibility. Among these 79 full-text articles, 53 articles were excluded for only meta-analysis and drug experiments on animals and without case-control and original genotype frequencies, finally 26 eligible studies on the association between *NAT2* polymorphisms and lung cancer risk were included in our meta-analysis. Twenty-two studies involved phenotypes^11,12,18–21,23–26,29–38,43,44^ and 4 studies discussed about genotypes.^39–42^ Diverse genotyping methods were used, including polymerase chain reaction (PCR), PCR-restriction fragment length polymorphism (PCR-RFLP), TaqManSNP (TaqMan), arrayed primer extension.

### Meta-Analysis

**FIGURE 2:**
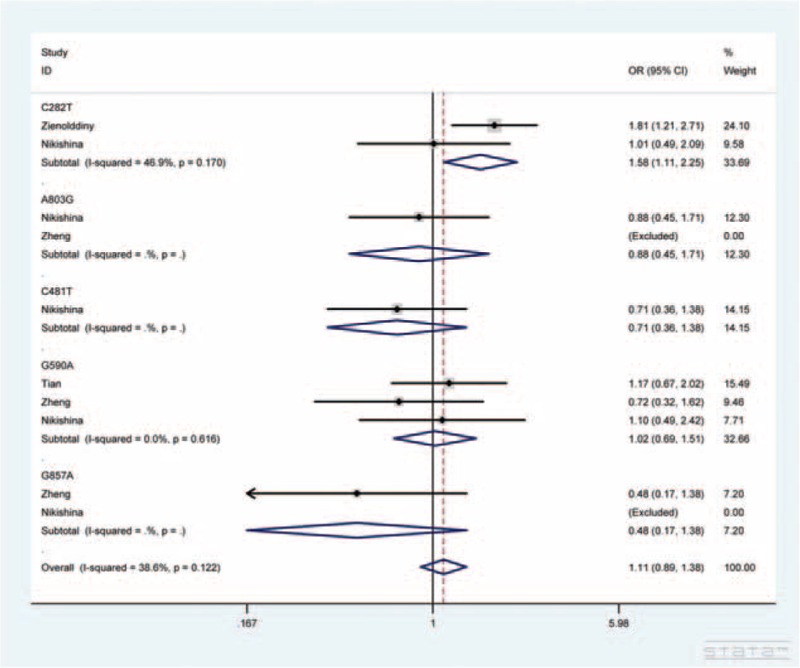
Forest plot of lung cancer susceptibility associated with *NAT2* C282T polymorphism under TT versus CC + TC genetic model. For each study, the estimates of OR and its 95% CI are plotted with square and a horizontal line. The area of the squares reflects the weight (inverse of the variance). The diamond represents the summary OR and 95% CI.

The main results are shown in Tables 3 and 4. Overall, with respect to phenotypes, the pooled ORs showed no significant association of *NAT2* polymorphisms with lung cancer susceptibility. In the subgroup analyses by ethnicity and source of control, there was still no significant association. In terms of genotypes, no obvious relationship was found between 5 SNPs in *NAT2* gene and lung cancer susceptibility. But increased risk of lung cancer was found in association with *NAT2* C282T polymorphism (TT vs. CC + TC: OR = 1.58, 95% CI = 1.11–2.25), as displayed in Figure 2.

**TABLE 3:**
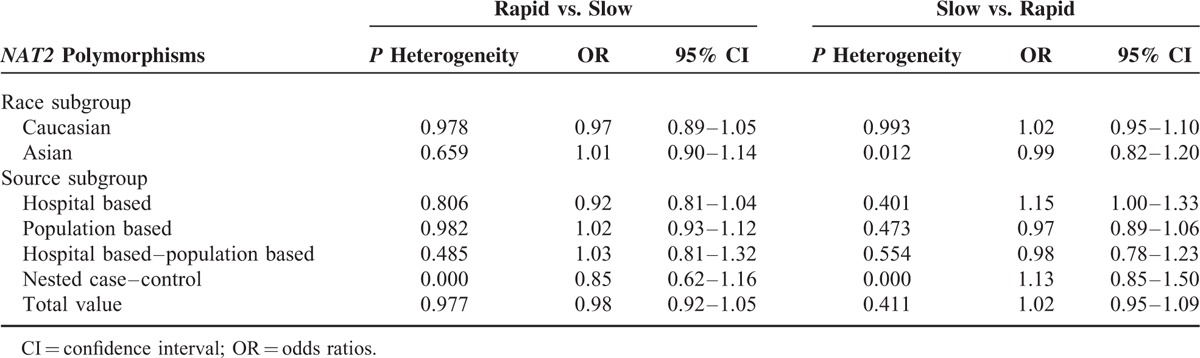
*NAT2* Polymorphisms With Phenotypes and Lung Cancer Risk

**TABLE 4:**
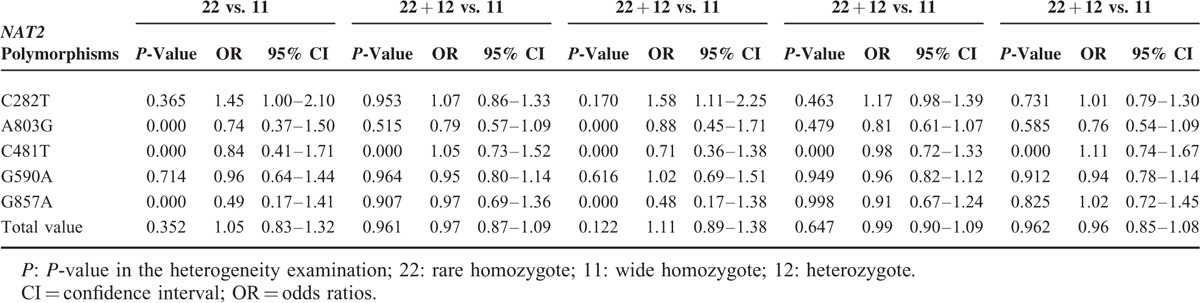
*NAT2* Polymorphisms With Genotypes and Lung Cancer Risk

### Sensitivity Analysis

Sensitivity analysis was carried out to evaluate the influence of each individual study on the pooled ORs. The recalculated ORs were not materially altered, suggesting our results were statistically steady.

### Publication Bias

**FIGURE 3:**
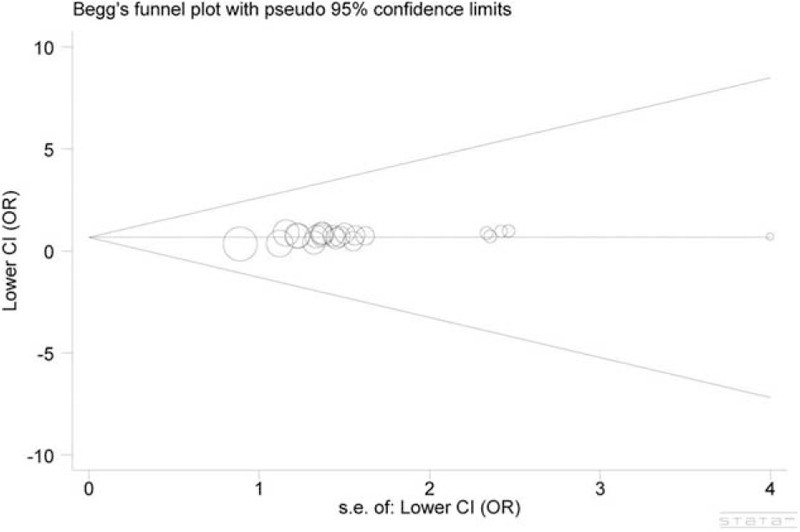
Begg funnel plot of publication bias test. Each point represents a separate study for the indicated association. Log(OR), natural logarithm of OR. Horizontal line, mean effect size.

Egger test and Begg funnel plot were performed to estimate the publication bias. The shape of the funnel plot did not indicate obvious asymmetry, as displayed in Figure 3. Additionally, result of Egger test did not show statistical evidence for bias (*P* = 0.805). Thus, there was no obvious publication bias and the results were credible.

## DISCUSSION

Both environmental and genetic factors are considered crucial in the etiology of lung cancer. The risk of lung cancer correlated with exposure to exogenous xenobiotics or endogenous substances may be modified by the genetic variation in metabolic detoxiﬁcation activities. Thus, the relevance of the *NAT2* polymorphisms to lung cancer risk is of particular importance.^45^

So far, the role of NAT2 acetylator status in lung cancer risk is unclear. Some epidemiological studies demonstrated that lung cancer susceptibility was not associated with NAT2 acetylator status.^18,25,28,30,35^ However, some investigators held the opinion that slow acetylator phenotype of *NAT2* polymorphisms was associated with increased risk of lung cancer.^20,24^ In our meta-analysis, there was no significant association between slow acetylator phenotype of *NAT2* polymorphisms and lung cancer risk. The disagreement may underlie differences in study population. Specifically, our study was based on Asians and Caucasians, whereas the studies of Sobti et al and Oyama et al were respectively performed in the North Indian population and Japanese population. With respect to rapid acetylator phenotype, Sorensen et al pointed out the NAT2 rapid acetylator phenotype seemed to be protective against lung cancer in light smokers but not among heavy smokers.^34^ Nevertheless, several research indicated rapid acetylator phenotype may contribute to increased risk of lung cancer.^23,36,37^

From the perspective of genotypes, to our knowledge, this is the second study definitely clarifying the association of *NAT2* C282T polymorphism with increased risk of lung cancer, which is not in accordance with Nikishina et al.^39^ The inconsistency may be on account of study population and sample size. In detail, our study included 1988 cases and 2411 controls among Asians and Caucasians, while the study of Nikishina et al was performed in only 122 cases and 167 controls among Caucasians living in Novosibirsk.

Some limitations in our study should be pointed out. First, in the subgroup analysis by ethnicity, our study was based on Asians and Caucasians, not considering other ethnic groups. Second, our study was not stratified by smoking status which is an important cause of lung cancer. Finally, lacking some original data of genotypes, the comprehensiveness and precision of association between *NAT2* polymorphisms and lung cancer may be influenced.

In conclusion, our meta-analysis demonstrated that TT genotype in C282T polymorphism among 5 SNPs in *NAT2* gene was a susceptibility factor for lung cancer. Additionally, the acetylator status of *NAT2* polymorphisms was not in association with lung cancer susceptibility. In the future, well-designed studies are required to give more comprehensive understanding of the association.
